# Current status and trends of machine learning applied in clinical research of gastric cancer from 2004 to 2023: global bibliometric and visual analysis

**DOI:** 10.3389/fonc.2025.1420517

**Published:** 2025-05-06

**Authors:** Xinyi Wang, Chao Wu, Siqing Yue, Mengyuan Zhou, Enba Zhuo, Xin Wu, Yafen Wang, Bangjie Chen, Fan Wang

**Affiliations:** ^1^ Department of Radiation Oncology, The First Affiliated Hospital of Anhui Medical University, Hefei, Anhui, China; ^2^ Department of Oncology, The First Affiliated Hospital of Anhui Medical University, Hefei, Anhui, China; ^3^ Inflammation and Immune Mediated Diseases Laboratory of Anhui Province, Anhui Institute of Innovative Drugs, School of Pharmacy, Anhui Medical University, Hefei, Anhui, China; ^4^ Institute for Liver Diseases of Anhui Medical University, Hefei, Anhui, China; ^5^ Department of Anesthesiology, The First Affiliated Hospital of Anhui Medical University, Hefei, Anhui, China; ^6^ First Clinical Medical College, Anhui Medical University, Hefei, Anhui, China

**Keywords:** bibliometrics, gastric cancer, machine learning, global trends, diagnosis, treatment

## Abstract

**Background:**

Gastric cancer is a serious disease that threatens human life; early diagnosis and treatment have been the focus of many studies. With advancements in imaging evaluation and machine learning, early detection and treatment of gastric cancer have become feasible. This study aimed to explore research trends and hotspots in the field of gastric cancer and machine learning through bibliometric analysis and to provide new insights for related clinical applications.

**Methods:**

Literature on gastric cancer and machine learning published from 2004 to 2023 was retrieved from the Web of Science database. Microsoft Excel 2019 was used for statistical analysis of influential articles, journals, authors, organizations, countries (regions), and co-citation references in this research domain. VOSviewer (version 1.6.16) and CiteSpace (version 5.8.R3) were utilized to visualize the corresponding data.

**Results:**

We analyzed and evaluated 425 articles authored by 2,899 researchers from 825 organizations across 52 countries (regions). The People’s Republic of China, the Chinese Academy of Sciences, and the University of the Chinese Academy of Sciences were identified as leaders in this field. The article “Genome-wide cell-free DNA fragmentation in patients with cancer,” published in *Nature*, was the most frequently cited work. The diagnosis and treatment of gastric cancer have consistently been research hotspots, with a shift in focus from laboratory-based studies to clinical applications. This trend highlights the transition from etiology-oriented research to studies emphasizing treatment and practical applications.

**Conclusions:**

This study offers a comprehensive visual analysis of research on gastric cancer and machine learning, representing the most detailed bibliometric study in this domain. With the continuous advancement of research, artificial intelligence-assisted early diagnostic methods for gastric cancer and corresponding treatment strategies may emerge as a pivotal direction for future research in this area.

## 1 Introduction

Gastric cancer is the fifth most common malignancy worldwide and has a high mortality rate (1, 2). In recent years, the incidence of gastric cancer among younger populations (aged <50 years) has been gradually increasing (3). Owing to the asymptomatic nature of early gastric cancer, most cases are diagnosed at advanced stages. Currently, systemic chemotherapy is the primary treatment for metastatic gastric cancer, with the median overall survival of patients receiving conventional chemotherapy remaining below 12 months (4, 5). Consequently, research on the mechanisms underlying gastric cancer, as well as its early diagnosis, individualized treatment, and precise evaluation of disease progression and prognosis, is crucial.

Machine learning is an emerging and promising field for integrating large and complex datasets (6). The rapid expansion of data in biology, especially in clinical medicine, has driven the application of machine learning in medical research. By incorporating computer science, mathematics, and statistics, machine learning addresses medical challenges, enabling the recording of biological information and supporting auxiliary functions, such as disease detection and predictive modeling (7, 8). Gastric cancer, characterized by its complex etiology, substantial patient burden, and frequent late detection, poses serious threats to human life and health. Therefore, early detection, diagnosis, treatment, and prognosis evaluation are critical (9). Machine learning models based on specific characteristics can effectively predict patient prognosis and treatment responses, aiding in the development of personalized treatment plans (10, 11). By analyzing imaging and optical spectra of thousands of patients with gastric cancer across various stages and classifications, early diagnosis and intervention become possible, providing more comprehensive recommendations for subsequent medical plans (12).

Extensive research has been conducted on gastric cancer and machine learning, with a wealth of documents and data playing an indispensable role (13). The growing availability of online databases and advancements in analytical software have increased the prominence of bibliometric approaches. Bibliometric analysis provides a scientific and quantitative method for evaluating literature (14). Visualizing medical research on gastric cancer using bibliometric tools enables researchers to quickly identify development trends, target high-quality authors and institutions, pinpoint influential research topics and credible studies, and accelerate scientific innovation (15). Tools, such as VOSviewer and CiteSpace, facilitate the generation of visual representations (16). In this study, we assessed the latest technological advancements in gastric cancer and machine learning research from 2004 to 2023 and employed bibliometric analysis to forecast future research directions. Our objective was to identify the research focus and key hotspots in this field, predict emerging trends, provide reference frameworks for understanding the mechanisms of gastric cancer, and contribute new scientific evidence for its early diagnosis and treatment in clinical practice.

## 2 Materials and methods

### 2.1 Source, retrieval, and filtering of data

**Figure 1:**
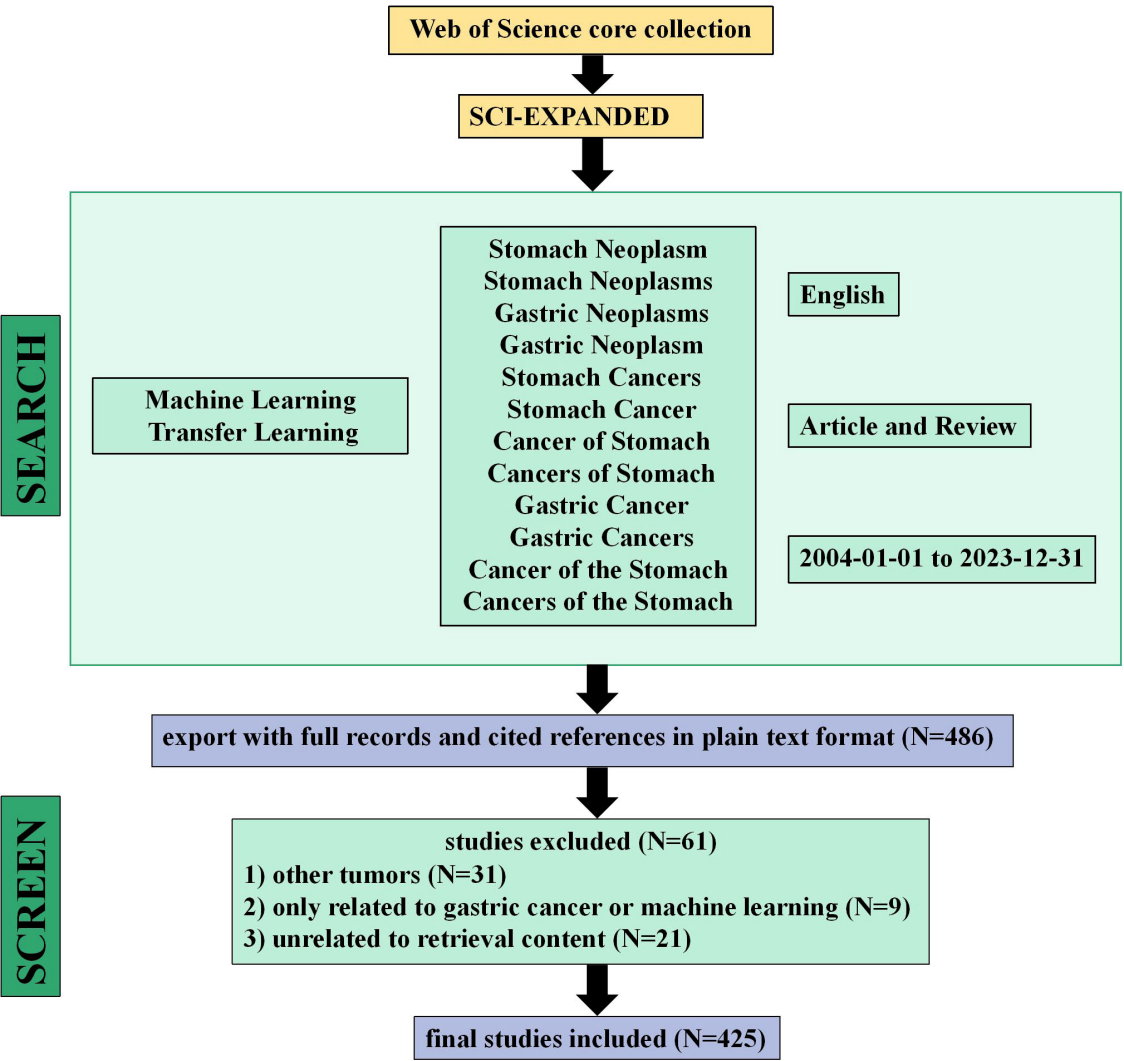
Flow diagram of the screening process related to gastric cancer and machine learning.

We retrieved the literature included in this study from the Web of Science (WoS) Core Collection as of December 31, 2023. As the world’s largest and most interdisciplinary academic information resource library, WoS is widely accepted by researchers and is regarded as the most suitable digital literature database for bibliometric analysis (17). Since the WoS Core Collection covers multiple disciplinary areas, we selected SCI-EXPANDED as the index. The search strategy employed the subject term “advanced search” method. The final search terms were as follows: (((TS=(Stomach Neoplasm OR Stomach Neoplasms OR Gastric Neoplasms OR Gastric Neoplasm OR Stomach Cancers OR Stomach Cancer OR Cancer of Stomach OR Cancers of Stomach OR Gastric Cancer OR Gastric Cancers OR Cancer of the Stomach OR Cancers of the Stomach)) AND TS=(Machine Learning OR Transfer Learning)) AND DT=(Article OR Review)) AND LA=(English). This strategy incorporated articles and reviews as document types and retrieved only English-language papers. We initially retrieved 480 articles. After screening, 425 articles were included. The search and screening process is illustrated in **Figure 1**. All data were downloaded from WoS in “plain text” format, including complete records and cited references. As this study did not involve animals or experiments, ethical approval was not required. The two authors (Bang-jie Chen and Chao Wu) independently conducted all search and screening work. Any disagreements were resolved through discussion or by consulting a third author (Xin-yi Wang).

### 2.2 Analysis of data and bibliometric software

To analyze the overall distribution characteristics, we used Microsoft Excel 2019 to summarize the top 10 most influential documents, journals, authors, organizations, countries (regions), and references in the field of machine learning research applied to gastric cancer. We used VOSviewer (version 1.6.16) and CiteSpace (version 5.8.R3) for visual analysis. These tools filter and sort data, display network visualization maps, and overlay visualization by constructing nodes and edges. CiteSpace produces more complex images than VOSviewer and is capable of dual-map overlay analysis, timeline and cluster view analysis, and burst detection to identify research trends in specific periods and fields.

## 3 Results

### 3.1 Overall distribution characteristics analysis

The 425 papers analyzed in this study were authored by 2,899 individuals from 825 organizations in 52 countries (regions), published in 219 journals, and cited in 16,512 references.

#### 3.1.1 Analysis of the publications distribution

**Figure 2:**
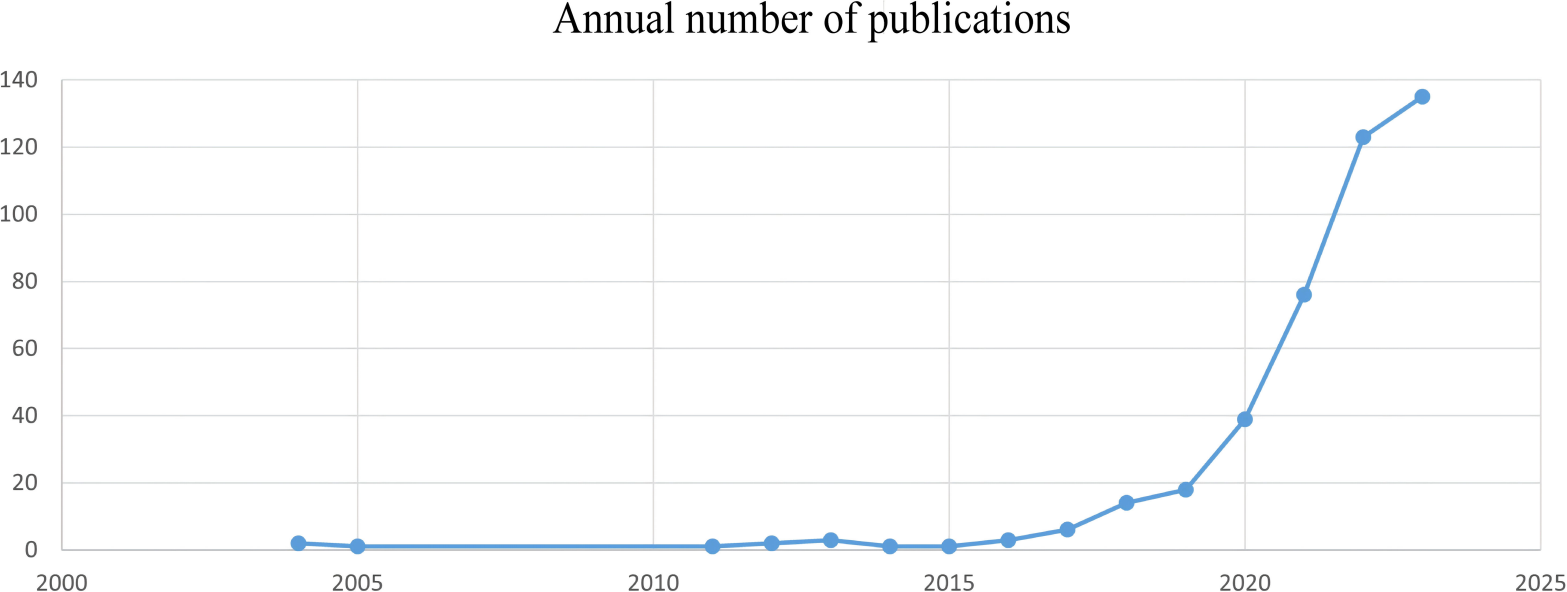
Annual number of published gastric cancer and machine learning studies, 2004–2023.

After screening, the cumulative number of studies on gastric cancer and machine learning published from 2004 to 2023 was determined to be 425. Research trends were analyzed by filtering the publication timelines, as shown in **Figure 2**. From 2004 to 2015, this research field received little attention, with a relatively small number of papers published annually. Between 2015 and 2019, the growth rate of publications was modest. However, from 2019 to 2023, the number of publications increased significantly, peaking at 135 papers in 2023. **Table 1** lists the top 10 cited documents in the study of gastric cancer and machine learning, most of which are located in the Q1 division. Among these, the paper “Genome-wide cell-free DNA fragmentation in patients with cancer,” published in *Nature*, was cited most frequently (18).

**Table 1:** Top 10 cited documents for the study of ML and GC.

Rank	Title	Citations	Journal	IF*	Quartile in category	Author	Year
1	Genome-wide cell-free DNA fragmentation in patients with cancer	554	*Nature*	64.8	Q1	Cristiano	2019
2	A deep learning-based multi-model ensemble method for cancer prediction	259	*Computer Methods and Programs in Biomedicine*	6.1	Q1	Xiao	2018
3	Application of convolutional neural network in the diagnosis of the invasion depth of gastric cancer based on conventional endoscopy	194	*Gastrointestinal Endoscopy*	7.7	Q1	Zhu	2019
4	Deep convolutional neural networks for automatic classification of gastric carcinoma using whole slide images in digital histopathology	166	*Computerized Medical Imaging and Graphics*	5.7	Q1	Sharma	2017
5	Artificial intelligence as the next step toward precision pathology	144	*Journal of Internal Medicine*	11.1	Q1	Acs	2020
6	Cancer Diagnosis Through IsomiR Expression with Machine Learning Method	106	*Current Bioinformatics*	4	Q1	Liao	2018
7	Circular RNAs and complex diseases: from experimental results to computational models	100	*Briefings in Bioinformatics*	9.5	Q1	Wang	2021
8	Plasmonic Alloys Reveal a Distinct Metabolic Phenotype of Early Gastric Cancer	98	*Advanced Materials*	29.4	Q1	Su	2021
9	Neopepsee: accurate genome-level prediction of neoantigens by harnessing sequence and amino acid immunogenicity information	86	*Annals of Oncology*	50.5	Q1	Kim	2018
10	Computer-Aided Gastrointestinal Diseases Analysis From Wireless Capsule Endoscopy: A Framework of Best Features Selection	79	*IEEE Access*	3.9	Q2	Khan	2020

*The impact factors (IF) of journals were obtained from the 2022 Web of Science Journal Citation Reports (JCR).

#### 3.1.2 Analysis of the journal distribution

**Figure 3:**
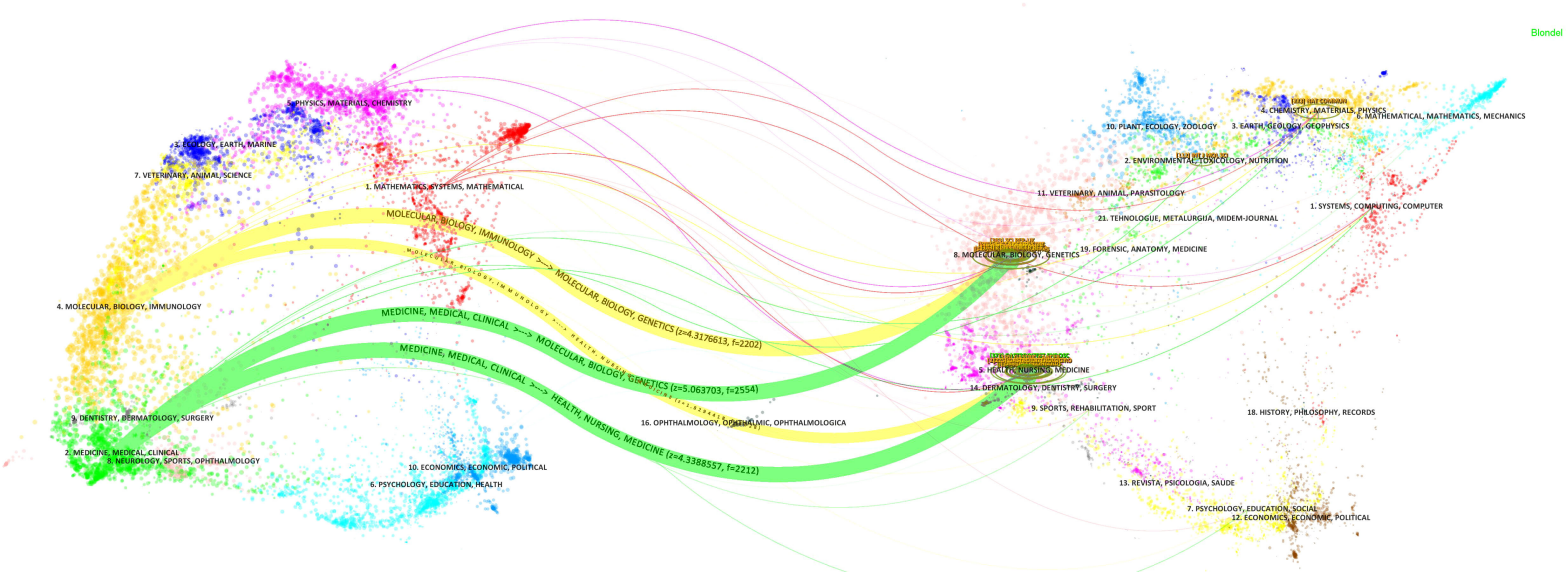
Dual-map overlay of gastric cancer and machine learning research-related journals.

The dual-map overlay analysis of journals revealed the distribution of citing and cited journals (**Figure 3**). The map on the left represents the disciplinary distribution of citing journals, whereas the map on the right illustrates citation relations among these journals. Points represent journals, and lines denote citation relations. The journals related to gastric cancer and machine learning span diverse fields, including medicine, clinical health, nursing, molecular biology, immunology, chemistry, pharmacology, and kinesiology. **Table 2** lists the 10 most influential journals in this field. The top three journals are *Nature*, *Computer Methods and Programs in Biomedicine*, and *Gastrointestinal Endoscopy*.

**Table 2:** Top 10 most influential source journals for the study of ML and GC.

Rank	Journal	Citations	Documents	Average Citation	Country(Region)	IF*	Quartile in category
1	*Nature*	554	1	554	England	64.8	Q1
2	*Computer Methods and Programs in Biomedicine*	306	4	76.5	Ireland	6.1	Q1
3	*Gastrointestinal Endoscopy*	280	4	70	United States	7.7	Q1
4	*Computerized Medical Imaging and Graphics*	166	1	166	United States	5.7	Q1
5	*Nature Communications*	155	5	31	England	16.6	Q1
6	*Journal of Internal Medicine*	144	1	144	England	11.1	Q1
7	*Briefings in Bioinformatics*	133	7	19	England	9.5	Q1
8	*Scientific Reports*	123	16	7.7	England	4.6	Q2
9	*Computers in Biology and Medicine*	117	6	19.5	United States	7.7	Q1
10	*Endoscopy*	116	2	58	Germany	9.3	Q1

*The impact factors (IF) of journals were obtained from the 2022 Web of Science Journal Citation Reports (JCR).

### 3.2 Co-authorship analysis

#### 3.2.1 Analysis of authors and cooperation

**Figure 4:**
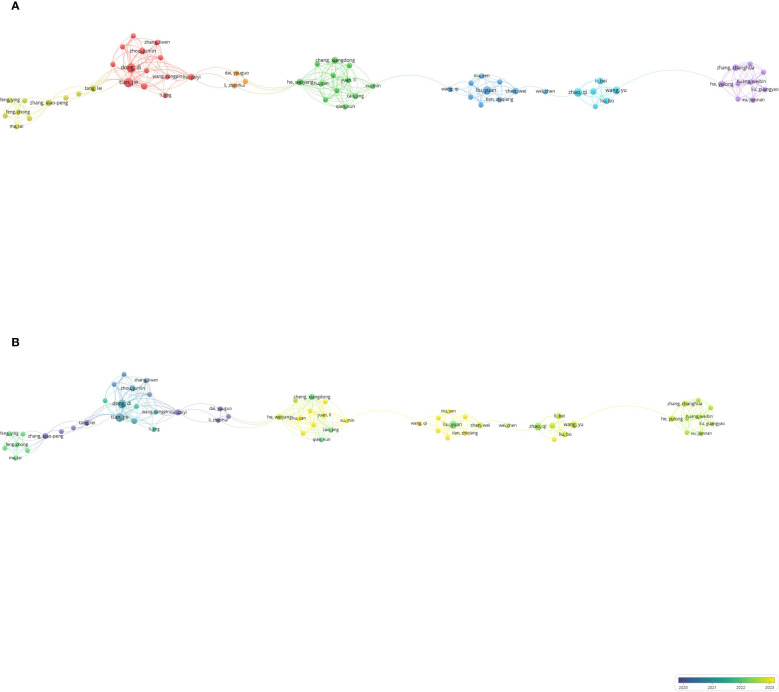
Co-authorship analysis of influential authors in the field of gastric cancer and machine learning. **(A)** Network visualization map of collaborations among the first 290 authors. **(B)** Overlay visualization map of collaborations among the first 290 authors.

A total of 2,899 authors have published literature in this field, with 290 contributing at least two articles. Among these, 63 authors collaborated with one another. The network visualization map indicates that Dong and Di have the most co-authors, followed by Tian Jie, He, and Weiyang, who exhibit strong connections with other authors in the network (**Figure 4A**). As this research field is relatively new, many authors began publishing their articles around 2022 and 2023. Authors who initiated research in the early stages include Zhang, Xiao-Peng, Wang, Zhi-Long, and Sunying-Shi (**Figure 4B**). Notably, in 2011, three authors co-published a study using machine learning to evaluate lymph node metastasis in gastric cancer (19). **Table 3** lists the top 10 most influential authors; Dong, Di, and Tian Jie lead in terms of the number of documents, average citations, and collaborations.

**Table 3:** Top 10 most influential authors for the study of ML and GC.

Rank	Author	Documents	Citations	Average citation
1	Khan, Muhammad Attique	7	180	25.7
2	Dong, Di	6	166	27.7
3	Tian, Jie	6	166	27.7
4	Zhang, Yu-dong	6	101	16.8
5	Zhao, Qi	5	122	24.4
6	Kadry, Seifedine	5	115	23
7	Feng, Qiu-xia	5	82	16.4
8	Liu, Xi-sheng	5	82	16.4
9	Qi, Liang	5	82	16.4
10	Wu, Lianlian	5	71	14.2

#### 3.2.2 Analysis of institutions and cooperation

**Figure 5:**
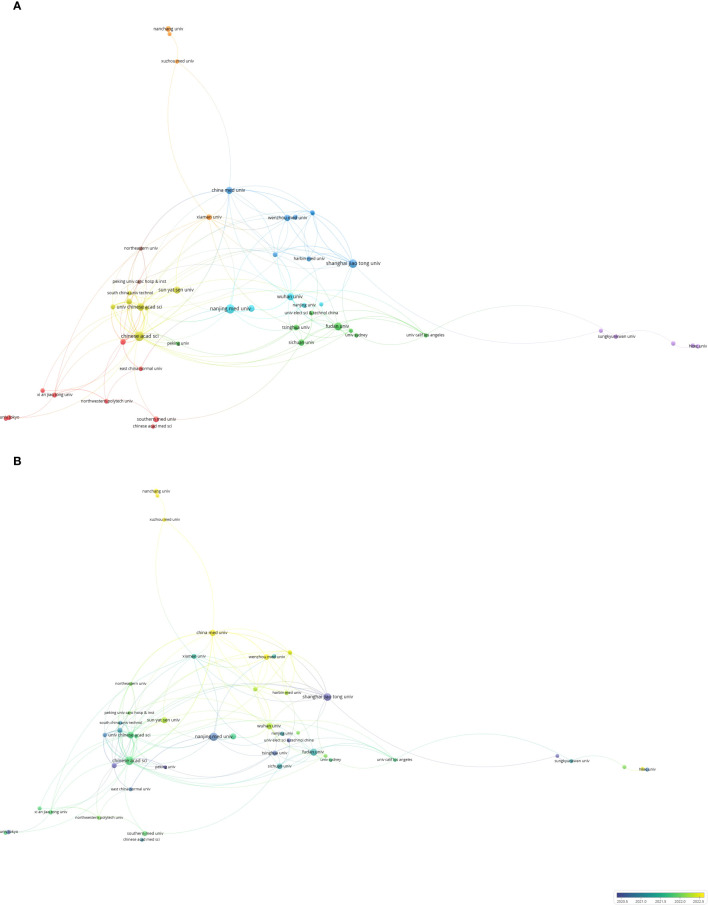
Co-authorship analysis of influential institutions in the field of gastric cancer and machine learning. **(A)** Network visualization map of collaborations among the first 53 institutions. **(B)** Network visualization map of collaborations among the first 53 institutions.

As shown in **Figure 5**, 825 institutions published articles, with 53 institutions contributing more than four articles. Among them, 48 institutions collaborated with one another. The Chinese Academy of Sciences and University of the Chinese Academy of Sciences emerged as the most collaborative institutions (**Figure 5A**). Additionally, the Chinese Academy of Sciences, Nanjing Medical University, and Shanghai Jiao Tong University have established influential institutional partnerships. Nanchang University, Soochow University, and Xuzhou Medical University have been the most prominent institutions in recent years, all located in the People’s Republic of China (**Figure 5B**). Among the top 10 institutions in terms of publication volume, nine are based in the People’s Republic of China (**Table 4**).

**Table 4:** Top 10 most influential organizations for the study of ML and GC.

Rank	Organization	Country (Region)	Documents	Citations	Average citation
1	Chinese Academy of Sciences	Peoples Republic of China	20	298	14.9
2	Nanjing Medical University	Peoples Republic of China	19	159	8.4
3	Shanghai Jiao Tong University	Peoples Republic of China	17	540	31.8
4	Fudan University	Peoples Republic of China	14	351	25.1
5	University of Chinese Academy of Sciences	Peoples Republic of China	13	209	16.1
6	Wuhan University	Peoples Republic of China	11	114	10.4
7	China Medical University	Peoples Republic of China	11	81	7.4
8	Sun Yat-sen University	Peoples Republic of China	11	68	6.2
9	Yonsei University	South Korea	10	203	20.3
10	Zhengzhou University	Peoples Republic of China	10	82	8.2

#### 3.2.3 Analysis of countries (regions) and cooperation

**Figure 6:**
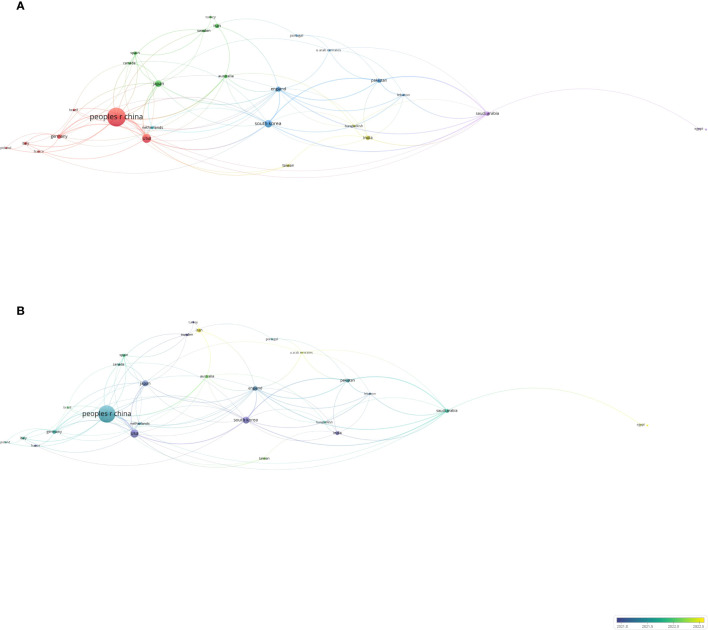
Co-authorship analysis of influential countries/regions in the field of gastric cancer and machine learning. **(A)** Network visualization map of collaborations among the first 27 countries/regions. **(B)** Network visualization map of collaborations among the first 27 countries/regions.

VOSviewer identified 52 countries (regions), with 27 publishing more than three articles and forming cooperative partnerships. The People’s Republic of China, the United States, England, and South Korea were the most prolific and extensive collaborators (**Figure 6A**). Temporally, scholars from Iran and Malaysia have only recently begun to focus on fields related to gastric cancer and machine learning (**Figure 6B**). **Table 5** lists the top 10 countries (regions) by publication volume, with the People’s Republic of China ranking first, substantially surpassing the second-ranking country.

**Table 5:** Top 10 most influential countries (regions) for the study of ML and GC.

Rank	Country (Region)	Citations	Documents	Average citation
1	Peoples Republic of China	2805	247	11.4
2	USA	1543	57	27.1
3	South Korea	681	40	17.0
4	Japan	373	31	12.0
5	England	239	18	13.3
6	Germany	306	15	20.4
7	Saudi Arabia	168	15	11.2
8	Iran	53	15	3.5
9	India	235	14	16.8
10	Pakistan	198	12	16.5

### 3.3 Analysis of high-frequency keywords

**Figure 7:**
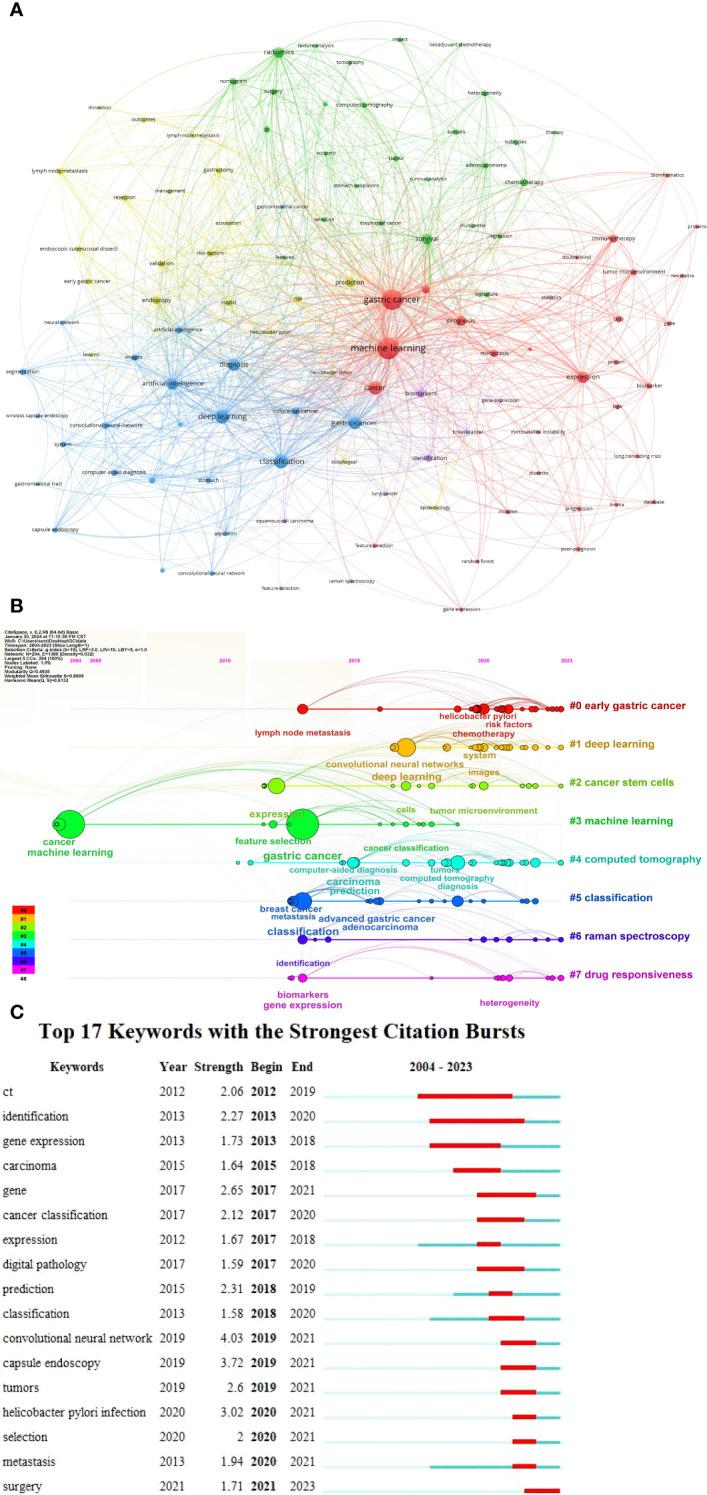
Visualization analysis of co-occurrence keywords in the field of gastric cancer and machine learning. **(A)** Network visualization map of 110 high-frequency keywords. **(B)** Timeline and clustering view map of all keywords. **(C)** Seventeen keywords with the strongest citation bursts.

Among the 1,807 keywords identified, we selected 110 high-frequency words with occurrences greater than or equal to six (**Figure 7A**). These 110 high-frequency keywords were grouped into five clusters: Cluster 1 (red): Gastric cancer diagnosis, including topics, such as cancer development, tumor immunology, tumor genes, and tumor markers. Cluster 2 (green): Survival analysis, encompassing nomograms, surgery, and chemotherapy. Cluster 3 (blue): Deep learning and artificial intelligence (AI), including applications, such as capsule endoscopy, convolutional neural networks, computer-aided diagnosis, and intelligent recognition. Cluster 4 (yellow): Risk factors, such as gastrectomy, *Helicobacter pylori* infection, lymph node metastasis, and early gastric cancer. Cluster 5 (purple): Miscellaneous topics. **Figure 7B** presents the timeline and clustering view of all keywords. Early gastric cancer, deep learning, cancer stem cells, machine learning, CT, classification, Raman spectroscopy, and drug responsiveness dominate the field. Raman spectroscopy and CT are noted as auxiliary tools for gastric cancer examination and prognosis, whereas pathological diagnosis determines tumor stage and classification. Early gastric cancer generally has a favorable prognosis; however, drug intolerance or the presence of cancer stem cells may result in poor long-term outcomes, including distant tumor metastasis. Deep learning, as a subset of machine learning, has gained widespread attention since 2015, underscoring its unique importance. **Figure 7C** illustrates the 17 keywords with the strongest citation bursts between 2012 and 2023. In the early years, the small number of articles precluded the formation of burst keywords. Before 2017, research focused primarily on genes, digital pathology, and tumor tissue typing, reflecting an emphasis on the microscopic aspects of gastric cancer. Since 2019, neural networks, capsule endoscopy, and surgical treatment have emerged as dominant topics, with research shifting toward tumor diagnosis and treatment.

### 3.4 Analysis of co-cited references

**Figure 8:**
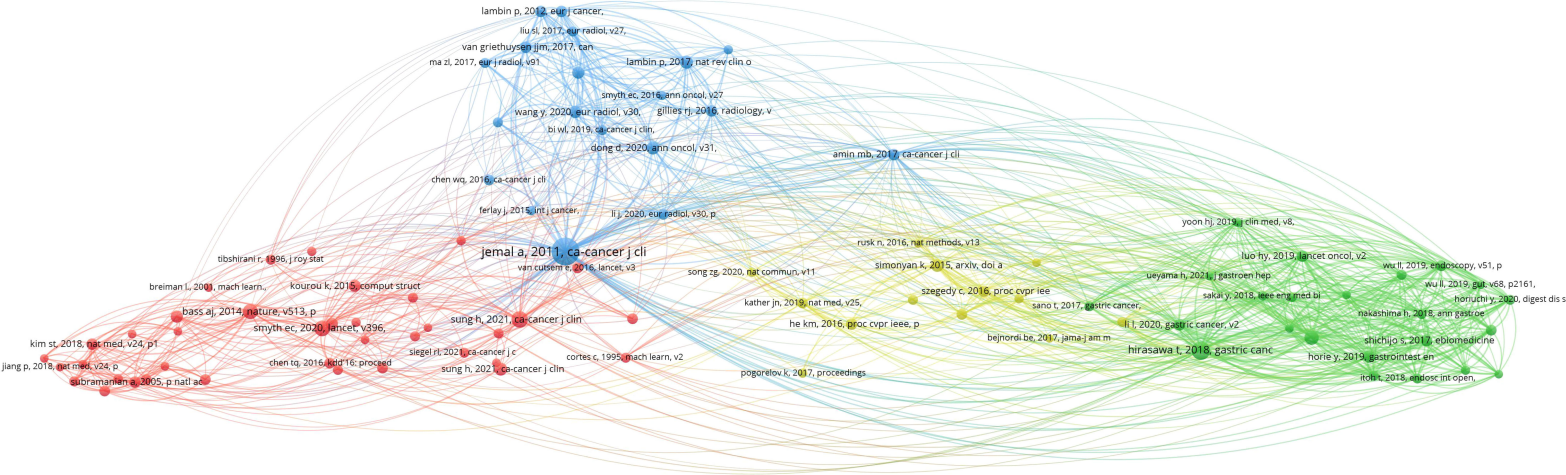
Visualization analysis of co-cited references in the field of gastric cancer and machine learning.

**Figure 8** depicts the 93 most highly cited articles, each with at least 10 citations. The network visualization map reveals that the majority of cited references originate from top-tier journals, particularly in disciplines, such as immunology and oncology. The article “Global cancer statistics 2018: GLOBOCAN estimates of incidence and mortality worldwide for 36 cancers in 185 countries,” authored by Jemal et al. and published in *CA: A Cancer Journal for Clinicians* in 2018, holds a central position among co-cited references. It has been cited 94 times, ranking first (**Table 6**) (20). This seminal article introduced the concept of applying AI to gastric cancer and laid a foundational theoretical framework for subsequent research on machine learning in this field over the next decade. These highly cited references underscore the flow of research hotspots and provide essential theoretical support for the evolving frontier fields of gastric cancer and machine learning.

**Table 6:** Top 10 co-citation references for the study of ML and GC.

Rank	Title	Citations	Author	Year	Journal	IF*	Quartile in category
1	Global cancer statistics 2018: GLOBOCAN estimates of incidence and mortality worldwide for 36 cancers in 185 countries	94	Jemal, Ahmedin	2018	*CA-A Cancer Journal for Clinicians*	254.7	Q1
2	Global cancer statistics 2020: GLOBOCAN estimates of incidence and mortality worldwide for 36 cancers in 185 countries	54	Sung, Hyuna	2021	*CA-A Cancer Journal for Clinicians*	254.7	Q1
3	Application of artificial intelligence using a convolutional neural network for detecting gastric cancer in endoscopic images	41	Hirasawa, Toshiaki	2018	*Gastric Cancer*	7.4	Q1
4	Gastric cancer	31	Smyth, Elizabeth C.	2020	*Lancet*	168.9	Q1
5	Comprehensive molecular characterization of gastric adenocarcinoma	27	Bass, Adam J.	2014	*Nature*	64.8	Q1
6	Application of convolutional neural network in the diagnosis of the invasion depth of gastric cancer based on conventional endoscopy	26	Zhu, Yan	2019	*Gastrointestinal Endoscopy*	7.7	Q1
7	Very Deep Convolutional Networks for Large-Scale Image Recognition	21	Simonyan, Karen	2015	*Arxiv*	–	Q1
8	Deep learning radiomic nomogram can predict the number of lymph node metastasis in locally advanced gastric cancer: an international multicenter study	20	Dong, D.	2020	*Annals of Oncology*	50.5	Q1
9	Radiomics: the bridge between medical imaging and personalized medicine	20	Lambin, Philippe	2017	*Nature Reviews Clinical Oncology*	78.8	Q1
10	CT radiomics nomogram for the preoperative prediction of lymph node metastasis in gastric cancer	19	Wang, Yue	2020	*European Radiology*	5.9	Q1
10	Diagnostic outcomes of esophageal cancer by artificial intelligence using convolutional neural networks	19	Horie, Yoshimasa	2019	*Gastrointestinal Endoscopy*	7.7	Q1
10	Development and validation of an individualized nomogram to identify occult peritoneal metastasis in patients with advanced gastric cancer	19	Dong, D.	2019	*Annals of Oncology*	50.5	Q1
10	Molecular analysis of gastric cancer identifies subtypes associated with distinct clinical outcomes	19	Cristescu, Razvan	2015	*Nature Medicine*	82.9	Q1

*The impact factors (IF) of journals were obtained from the 2022 Web of Science Journal Citation Reports (JCR).

## 4 Discussion

Bibliometrics provides a comprehensive framework for summarizing research from the past to the present, identifying highly productive journals, authors, institutions, and countries, as well as highlighting highly cited documents and references in a given field. It also aids in predicting future research directions and trends (21). While numerous articles have discussed the relation between machine learning and gastric cancer, the lack of a consolidated summary often limits the comprehensiveness and macro-level understanding of these works, making it difficult to discern a broad developmental framework. Our bibliometric analysis reviewed all English-language literature on gastric cancer and machine learning published in the WoS Core Collection over the past 20 years. This study highlighted notable trends and landmark articles in this area between 2004 and 2023. Machine learning has shown substantial potential in promoting the early diagnosis, treatment, and prognosis prediction of gastric cancer. By integrating the expertise of generations of clinicians with cutting-edge technologies, clinical diagnosis and treatment can become more digital, robotic, and precise (22).

Research at the intersection of machine learning and gastric cancer encompasses the fields of AI and clinical medicine. While clinical medicine, being an empirical science, relies on continuous data accumulation and is limited by manpower, machine learning offers constant updates and improvements. Multidisciplinary management and interdisciplinary collaboration are becoming essential in clinical medicine, and the thoughtful application of AI can enhance the feasibility and reliability of clinical research (23–25). Our analysis revealed that the People’s Republic of China leads the field in terms of citation numbers, as well as the contributions of top authors and institutions. The incidence of gastric cancer varies by region (4). The traditional Chinese diet, characterized by high protein, fat, and salt content, combined with hereditary factors and familial dietary habits, has contributed to China becoming a focal region for gastric cancer research. The integration of AI technologies, such as machine learning, has solidified China’s position as a leader in this domain. Developed countries, including the United States, the United Kingdom, and Germany, benefit from more established and comprehensive medical systems for early cancer screening. These nations are characterized by high levels of collaboration and research output. Notably, the 10 most influential journals in the field of machine learning and gastric cancer originate from developed countries, reflecting the importance of stable social structures and robust research funding in fostering innovation and high-quality publications. Leading institutions, such as the Chinese Academy of Sciences, the University of the Chinese Academy of Sciences, Nanjing Medical University, and Shanghai Jiao Tong University, have played pivotal roles in advancing research on gastric cancer and machine learning. These institutions leverage comprehensive academic platforms to integrate AI with clinical medicine. Their contributions include predictive models for lymph node metastasis, drug resistance, treatment efficacy evaluation, and metabolomics-based diagnostics and prognostics (26–28). However, most research is conducted by smaller groups, with limited global collaboration. To address this imbalance, organizing international academic conferences and fostering discussions and knowledge exchange is critical.

Highly cited authors and institutions began exploring the intersection of AI and clinical medicine at an early stage. Gastric cancer, with its large patient base, widespread use of endoscopy, and accessibility to extensive pathological specimens, offers a fertile ground for studies utilizing machine learning. These studies integrate substantial clinical data with AI methods, producing models of considerable diagnostic and treatment value. For instance, the highly cited article “Genome-wide cell-free DNA fragmentation in patients with cancer” describes the application of machine learning models that incorporate genome-wide fragmentation features across multiple cancer types, including gastric cancer (18). This study demonstrated a sensitivity range of 57% to >99% and specificity of 98% for detecting various cancers using machine learning models. It provides valuable insights into research methodologies and potential future directions for gastric cancer research in the era of AI.

By summarizing the high-frequency keywords by analogy and combining them with clinical pain points, we believe that early screening for gastric cancer is one of the most promising and popular areas of AI research in gastric cancer diagnosis and treatment (29). Moreover, in the literature collected over the past 20 years, early screening and diagnosis of gastric cancer have always been popular. The global hotspot for cancer treatment is to extend the survival of patients, and the most direct way to extend survival is through the early diagnosis and treatment of cancer. The traditional method for diagnosing gastric cancer involves the histopathological examination of biopsy specimens to identify the morphological features of malignant cells, which is both time-consuming and labor-intensive (30). There is an increasing need for imaging analysis, and the histological classification of gastric cancer is also increasing. Automatic segmentation of lesion areas is challenging in the assisted pathological diagnosis of gastric cancer (31). Our high-frequency keywords confirmed this change. In the early stages, the focus of research was more inclined toward studying the tumor mechanism; later, the focus shifted to pathology and tumor classification. In recent years, endoscopy and surgery have become popular research topics. The main research focus of combined machine learning for gastric cancer worldwide is early diagnosis and treatment, which represents the transformation from basic research to clinical application for gastric cancer.

AI has been applied in many medical imaging fields, such as endoscopy, pathology, and radiology (CT). AI can assist in automatic, precise, and rapid endoscopic and histological examinations by considering all relevant factors. Prateek S. and Toshiaki H. et al. summarized several controlled clinical trials to determine the added value of AI in the diagnostic process and found that the independent sensitivity of AI for endoscopic diagnosis of esophageal squamous cell carcinoma, Barrett’s esophagus-related tumors, and gastric cancer was between 83% and 93% (32, 33). Endoscopy plays a crucial role in the detection of gastric cancer because it allows endoscopists to directly observe cancerous areas (34). Accurate diagnosis of early gastric cancer using endoscopic images is urgently required to improve patient outcomes. However, the accuracy of traditional endoscopy is only 69–79% (35). Owing to the high workload involved in medical image analysis, experienced endoscopists may inevitably experience misdiagnosis and missed diagnoses (36). Therefore, AI, through machine learning methods, integrates traditional endoscopic images for further analysis and assistance, thereby improving the accuracy of clinical diagnoses (37). A study conducted in China in 2019 used the GRAIDS method to analyze 1,036,496 endoscopic images, improving the accuracy of the clinical endoscopist’s diagnosis of gastric cancer to 97.7% (38). Similarly, a 2019 study in Japan, based on deep neural networks, analyzed 107,284 endoscopic sample images and achieved a kappa value of 0.27 (39). These studies demonstrate that the diagnostic accuracy of early gastric cancer under endoscopy can be significantly improved through various algorithm models using machine learning. In the field of pathology, Jakob et al. showed that machine learning can predict microsatellite instability in tissue slices (40). Acs et al. reviewed several breakthrough studies, indicating that the application of machine learning in pathology has significantly improved lymph node metastasis detection and breast cancer Ki67 scores and has proved that it can predict the status of some molecular markers in gastric cancer according to standard HE slices (34–41).

In addition to endoscopy and pathology, imaging techniques, such as CT, have great application value in the clinical diagnosis of gastric cancer (42, 43). Accurate staging is a crucial step in determining the degree of tumor invasion. CT scans are routinely used for preoperative TNM staging of gastric cancer (44). However, the predictions based on subjective assessments by radiologists are not entirely convincing, with accuracy rates ranging from 50% to 70% (45). Dong et al. developed a deep learning algorithm model for radiographic images based on preoperative CT images to predict the number of metastatic lymph nodes in patients with locally advanced gastric cancer. The algorithm demonstrated considerable discrimination with Area Under Curve (AUC) values of 0.821, 0.797, and 0.822 for the primary, external, and international validation datasets, respectively (26). Additionally, Chen et al. explored the tumor immune microenvironment of gastric cancer. Unsupervised consensus clustering was applied to identify three immune subtypes with different immune cell infiltration components and molecular characteristics associated with distinct immune scores and prognoses. The immune subtypes were validated using two gastric cancer datasets and six pan-cancer datasets (46). This study highlights the potential of machine and deep learning to explore tumor immune microenvironments and immune subtypes, assisting in the strategic development of immunotherapies for gastric cancer. A flexible machine learning model requires a large amount of well-annotated data for training, validation, and testing, and studies with small sample sizes are prone to measurement errors (47). Yang et al. used machine learning analysis to reveal a 10-metabolite GC diagnostic model, which had a sensitivity of 0.905, compared to the traditional sensitivity of 0.40. Additionally, AI can categorize patients into different risk groups for early diagnosis and treatment of gastric cancer (48). With the advancement of medical imaging techniques, such as endoscopy and pathology, the continuous generation of large amounts of data can assist doctors in clinical diagnosis and decision-making.

After analyzing the articles studied, resection was recommended as the treatment method for early gastric cancer, whereas adjuvant chemotherapy and targeted molecular therapy were recommended for patients with advanced gastric cancer (49). As one of the earliest surgeries for gastric cancer, open surgery plays an important role in its treatment. Traditional surgical operations, such as subtotal gastrectomy and esophagojejunostomy, can significantly reduce the tumor load in patients, achieve radical curative effects for early gastric cancer, prolong life, and improve the quality of life of patients with advanced multiple metastases (50). Robotic gastrectomy has a shorter duration, less intraoperative blood loss, and fewer postoperative complications than those of laparoscopic gastrectomy. However, robotic surgery is not yet popular, and for most patients, a single surgical treatment is not the best plan; it is often necessary to combine chemotherapy with other drugs before or after surgery (51, 52). Adjuvant immunotherapy has also been introduced into perioperative treatment plans (53). AI methods that simulate human cognitive functions are adept at processing and analyzing large amounts of data using computers, making them useful for gastroenterologists in clinical diagnosis and decision-making. Researchers have explored the application of AI methods in resection surgery, chemotherapy, and selection of molecular drugs. For example, in 2020, Yang et al. used deep learning methods to train 1,244 gastric cancer tissue images and videos of patients treated with Endoscopic Submucosal Dissection, providing clinicians with assistance in surgical treatment decisions (54). The use of clinicopathological characteristics, CT, immunohistochemical staining, and lymph node WSIs for prognostic treatment demonstrates the potential of AI in different gastric cancer treatment practices.

Most patients with gastric cancer are diagnosed at an advanced stage, and radical surgery is not feasible (55). Systemic chemotherapy is recommended to prolong survival; however, tumor responses to monotherapy and combination chemotherapy vary among individuals. In recent years, with a deeper understanding of the molecular basis of tumors, targeted therapy has become an effective treatment option (56). Preoperative or postoperative histopathological examinations or genomic analyses are used to classify gastric cancer into different subtypes, aiding in the selection of molecular-targeted therapies. Joo et al. proposed a deep learning model to predict drug responses based on half-maximal inhibitory concentration (IC50) (57). The genomic and molecular features of cancer cells are spliced into an input vector for prediction. Researchers have also explored AI methods for predicting digital pathologies. Traditionally, pathologists calculate the positive cells in selected fields of view and classify them into different grades for prediction. However, their accuracy is affected by subjectivity and observer variation; thus, their accuracy is affected (58). Meier et al. developed an assumption-free deep learning model to predict risks based on immunohistochemically stained tissue microarrays (59).

Through bibliometric analysis, we found that the future research hotspots of gastric cancer lie in early diagnosis, early treatment, and the prediction of prognosis and survival. AI-assisted automatic endoscopic and pathological histology recognition under a microscope can improve the sensitivity of early diagnosis. Simultaneously, AI-assisted CT and other techniques can be used to assess metastasis and infiltration of gastric cancer based on a large amount of analytical data, thereby guiding clinicians in formulating individualized treatment plans and predicting patient survival. Machine learning is becoming increasingly important for promoting clinical diagnosis and treatment. Bibliometrics should be widely used as a research tool to analyze various diseases.

Our study has several strengths. First, this is a rare article that investigates the relation between machine learning and gastric cancer through bibliometric analysis, summarizing research hotspots and analyzing future research trends. It collects the majority of articles published over the past 20 years since the introduction of the Internet and conducts comprehensive and largescale research. Second, bibliometric analysis is time-saving, efficient, and economical, avoiding the need for the extensive manpower and resources required in normal experiments. In addition, the published literature has a high level of research quality and authenticity, making the research findings more credible.

However, our study has some limitations. First, although the WOS covers the majority of published articles, it inevitably misses some literature, and our selection criteria limited us to English-language articles. Second, the literature itself is affected by publication bias, with positive results more likely to be published than statistically insignificant negative results. Additionally, some recent groundbreaking papers may have had less impact than older papers and require further testing.

This study conducted a visual analysis of research on machine learning and gastric cancer, making it the most detailed and comprehensive bibliometric study in this field to date. The People’s Republic of China has had the most significant impact in this area. Developing countries are committed to becoming more innovative and must strengthen their cooperation with advanced institutions and countries (regions). Machine learning is one of the most important branches of AI and represents a pattern recognition and automation approach based on extensive data and algorithms. Machine learning can significantly improve the accuracy of early diagnosis and prognosis prediction of gastric cancer in existing clinical settings. However, current methods also face challenges, such as data scarcity and poor interpretability, which can be addressed through data regularization and advanced algorithms. Additionally, by developing multimodal and cross-modal algorithms and improving the model evaluation and clinical application processes, we aim to build more clinically useful AI application models for gastric cancer.

## Data Availability

Publicly available datasets were analyzed in this study. This data can be found here: https://www.webofscience.com/wos/.
